# Critical NIH Resources to Advance Therapies for Pain: Preclinical Screening Program and Phase II Human Clinical Trial Network

**DOI:** 10.1007/s13311-020-00918-2

**Published:** 2020-09-02

**Authors:** Smriti Iyengar, Sarah A. Woller, Rebecca Hommer, Jennifer Beierlein, Clinton B. Wright, Amir P. Tamiz, Barbara I. Karp

**Affiliations:** 1 Division of Translational Research, National Institutes of Health, National Institute of Neurological Disorders and Stroke, 6001 Executive Blvd, Bethesda, MD 20852-9535 USA; 2 Division of Clinical Research, National Institutes of Health, National Institute of Neurological Disorders and Stroke, 6001 Executive Blvd, Bethesda, MD 20852-9535 USA

**Keywords:** Pain, Therapeutics, Non-opioid, Screening, Clinical trials

## Abstract

Opioid-related death and overdose have now reached epidemic proportions. In response to this public health crisis, the National Institutes of Health (NIH) launched the Helping to End Addiction Long-term Initiative^SM^, or NIH HEAL Initiative^SM^, an aggressive, trans-agency effort to speed scientific solutions to stem the national opioid public health crisis. Herein, we describe two NIH HEAL Initiative programs to accelerate development of non-opioid, non-addictive pain treatments: The Preclinical Screening Platform for Pain (PSPP) and Early Phase Pain Investigation Clinical Network (EPPIC-Net). These resources are provided at no cost to investigators, whether in academia or industry and whether within the USA or internationally. Both programs consider small molecules, biologics, devices, and natural products for acute and chronic pain, including repurposed and combination drugs. Importantly, confidentiality and intellectual property are protected. The PSPP provides a rigorous platform to identify and profile non-opioid, non-addictive therapeutics for pain. Accepted assets are evaluated in in vitro functional assays to rule out opioid receptor activity and to assess abuse liability. *In vivo* pharmacokinetic studies measure plasma and brain exposure to guide the dose range and pretreatment times for the side effect profile, efficacy, and abuse liability. Studies are conducted in accordance with published rigor criteria. EPPIC-Net provides academic and industry investigators with expert infrastructure for phase II testing of pain therapeutics across populations and the lifespan. For assets accepted after a rigorous, objective scientific review process, EPPIC-Net provides clinical trial design, management, implementation, and analysis.

**Electronic supplementary material:**

The online version of this article (10.1007/s13311-020-00918-2) contains supplementary material, which is available to authorized users.

## Introduction

In 2011, the Institute of Medicine report on pain indicated that 100 million Americans report chronic pain [1], with 25 million reporting *daily* chronic pain [2]. This report emphasized chronic pain to be a public health crisis and increased attention on the treatment of acute pain, prevention of chronic pain, and chronic pain management, including disparities in pain and pain care. In response, Health and Human Services (HHS) established the Interagency Pain Research Coordinating Committee (IPRCC), which released the National Pain Strategy in 2016 [3]. This effort recognized the need for increased investment in basic and translational research on the biopsychosocial mechanisms leading to the development and maintenance of chronic pain, and the development of safe and effective pain treatments.

As the National Pain Strategy [3] was being unveiled, another public health crisis was emerging: the opioid crisis. Opioid use and deaths resulting from opioid overdoses have increased exponentially in the past several years [4]. While the factors contributing to the opioid crisis are multifaceted, one element common to both public health crises is the lack of effective, non-opioid pain therapeutics. As a response, the NIH HEAL Initiative was developed with the goals to 1) improve prevention and treatment strategies for opioid misuse and addiction and 2) enhance pain management [5].

Enhancing pain management is a complex task, requiring basic science, preclinical research and clinical trials. In response to the NIH HEAL Initiative call, the National Institute of Neurological Disorders and Stroke (NINDS) launched preclinical and clinical programs: The Preclinical Screening Platform for Pain (PSPP) and the Early Phase Pain Investigation Clinical Network (EPPIC-Net). In this report, we will describe the goals of these two programs, including the workflow and program guidance for each.

## Preclinical Screening Platform for Pain

The PSPP was developed with the goal of providing a platform to identify and profile non-opioid, non-addictive therapeutics and devices for pain. The framework for this program was modeled after the Epilepsy Therapy Screening Program (ETSP), which has been operating since 1975 [6]. The ETSP, formerly known as the Anticonvulsant Screening Program (ASP), has provided free testing in rodent seizure models run by a contract facility for more than 40 years, contributing to the identification of numerous new therapeutics (e.g., topiramate and lacosamide). PSPP similarly provides an efficient, rigorous, one-stop screening resource for potential pain therapeutics from worldwide academic institutions and industry at no cost to the PSPP participant. All screening is performed through a government contract in a blinded and confidential manner. In order to advance a comprehensive range of potential non-opioid treatments for pain, PSPP is set up to accept and screen small molecules, biologics, devices, and natural products. In contrast to the typical NIH grant funding cycle, PSPP evaluates and accepts assets for screening continuously, on an ongoing basis.

PSPP employs a multi-tier strategy for screening and profiling submitted assets. In tier 1, assets are evaluated in vitro*,* to rule out functional activity at opioid receptors and to assess abuse liability. Once it is clear that the asset does not have functional opioid activity or a potential for abuse liability, pharmacokinetic (PK) studies are performed to help inform further testing. PK analysis, including plasma and CNS tissue, will be conducted in male and female rats. Only promising assets are advanced to the next level. Significant effort is directed toward making sure the body of evidence generated in Tier 1 in conjunction with the information provided by the participant supports further testing in tier 2.

Tier 2 screening starts with *in vivo* side effect profile assessment for neurologic deficits using both an accelerated rotarod test and a modified Irwin test. All animal studies strictly follow animal welfare regulations [7] with approvals from the local Institutional Animal Care and Use Committee (IACUC). Next, assets are screened in acute/sub-chronic and chronic models of pain i.e. the plantar incision model and the L5/L6 spinal nerve ligation model, respectively.

If the proposed asset exhibits clear efficacy in tier 2 without side effect liability, it is then advanced for further evaluation in tier 3. At this level, test candidates are evaluated for abuse liability using a self-administration or conditioned place preference paradigm. Components of abuse liability (e.g., sedation or hyperlocomotion) are included in the modified Irwin screen. Assets are further examined in disease-specific pain models (e.g., osteoarthritis, chemotherapy-induced peripheral neuropathy, migraine, etc.). Together, these three tiers provide a robust initial preclinical assessment of efficacy and abuse liability.

The PSPP workflow is flexible and adaptable to allow for the best preclinical assessment of potential pain therapeutics. The PSPP program recognizes the limitations of current animal models (e.g., assessment of evoked responses, single dosing of test compounds) and aims to continuously validate and incorporate new models as well as new endpoints appropriate for assessing the many dimensions of pain. Importantly, an External Consultant Board (ECB) for PSPP provides independent input and oversight on scientific and strategic priorities and implementation, including input on selecting new models, developing and monitoring screening flows, prioritizing targets and mechanisms for interrogation, and establishing milestones for program progress. Additionally, in early 2019, NINDS held the “Critical Evaluation of Animal Pain Models for Therapeutics Development” workshop, which evaluated the current state of animal models in the field, the models that best represent the human pain condition, and models for future development. The outcome of that workshop provided an important resource for PSPP, both for the identification of new methods of evaluating pain and for informing the state of specific pain models where additional validation is necessary. Incorporating and validating new models will aid early drug development and translation to clinical use.

## Early Phase Pain Investigation Clinical Network

EPPIC-Net sits further along the pathway of development of pain therapeutics, focusing on phase 2 clinical trials. EPPIC-Net invites academic and industry partners with potential pain therapeutics to apply to EPPIC-Net. EPPIC-Net also accepts proposals for pain biomarker validation and deep phenotyping and incorporates a mandate to explore innovative trial designs. The application process for EPPIC-Net is designed to be quick and efficient, yet robust, encompassing three application/review stages that can be completed in 6–8 months. The first stage entails completion of a brief preliminary application, providing an overview of the proposed therapeutic, including information on preclinical and clinical studies completed, pain target, and trial concept. Following objective review, selected applicants move to the second “dossier” stage. At stage 2, an NIH contractor works with the applicant to prepare a dossier with in-depth information on the asset. For drugs, the dossier contains information on pharmacology and physiology. For devices, the dossier incorporates mode-of-action, mode-of-use and technical specifications. For both, citations of prior preclinical and clinical studies and information on NDA or IDE status are required. Dossiers undergo review by members of the same objective review panel as at stage 1. Selected dossier applicants are invited to stage 3 of the application process. At stage 3, the asset holder works with the EPPIC-Net Clinical Coordinating Center to design the clinical trial around the asset, along with a study timeline and proposed budget. Following a third objective review, studies are selected for implementation within EPPIC-Net. The Clinical Coordinating Center receives funding under NIH’s “Other Transaction (OT)” authority for the conduct of the trial within EPPIC-Net Specialized Clinical Research Centers. The asset holder maintains intellectual property rights to the asset and retains the right to move the asset forward in the development following completion of the phase 2 trial.

The EPPIC-Net infrastructure encompasses a Clinical Coordinating Center (CCC) at the Massachusetts General Hospital, a Data Coordinating Center at New York University, and 12 Specialized Clinical Research Centers throughout the USA. This network approach models other established NIH Clinical Trial Networks such as StrokeNet [8] and NeuroNEXT [9]. Cumulatively, EPPIC-Net provides access to pain clinicians and researchers with expertise across a broad range of pain conditions and to diverse pain patient populations. EPPIC-Net considers applications targeted to any pain condition with high unmet therapeutic need.

## Conclusion

PSPP and EPPIC-Net are aligned with both the 2017 Federal Pain Research Strategy’s call for safer, new, non-opioid analgesics and the NIH HEAL Initiative’s goal of accelerating the discovery and development of non-addictive pain treatments. The programs provide support for the development of a broad range of potential therapeutic approaches, incorporating rigorous preclinical testing and clinical trial paradigms to reinvigorate and encourage the development of new pain therapeutics.

## Electronic supplementary material

ESM 1 (PDF 1224 kb)
